# Medical and Physical Intelligence

**Published:** 1799-09

**Authors:** 


### MEDICAL AND PHYSICAL
### *INTELLIGENCE,*
### (Original and Selected.)

*As the subjects generally introduced in this department of our Journal have only a distant relation to the practice of the Medical Art, while they consist chiefly of miscellaneous articles, tending in some degree to mark the scientific progress in the* AUXILIARY BRANCHES OF PHYSIC, *we shall not hesitate to communicate to our readers any information transmitted by correspondents, who wish to aid our efforts, in attaining that desirable purpose.*

FROM a very ingenious and elaborate paper, originally written by Dr. MITCHILL of New York, and addressed to Dr. HOPE, the present Professor of Chemistry in the University of Edinburgh, *on the application of the doctrine of septic fluids, to explain some of the diseases of human teeth and bones*, we shall, under this head of our Journal, extract the part of Dr. Mitchill's disquisition, together with his experiments, relative to some of the morbid changes which the teeth and bones of animals undergo in the progress of life.

Dr. Mitchill aims at a scientific explanation of some of the alterations which bones suffer by disease; as the writers on surgery have scarcely ventured upon this inquiry. What he has aimed at in this respect is, to connect the facts related by the late Mr. HUNTER, of London, with those detailed by Mr. RUSSELL, of Edinburgh, by means of certain experiments and observations of his own. From the known disposition of oxygen to combine with septon, and from the septic acid, it appeared probable to Dr. Mitchill, that it would be formed occasionally in the human mouth, from the remains of food adhering to the teeth, settling between them, and corrupting there, If this were the case, this acid ought to unite with the calcareous earth of the teeth, and form the septite of lime, which might be washed away by the saliva, or possibly in some instances concrete upon the teeth themselves; and, if formed there, the acid might be expected to corrode the enamel, lay bare the bony part, and bring on a caries; or to incrust the outside, irritate the gums, and occasion soreness and bleedings.

In order to determine whether these effects actually took place, Dr. Mitchill procured from a dentist a quantity of the substance called the "tartar of the teeth," which he supposed might contain some septite of lime, and subjected it to a number of experiments.

Having some time before received complaints from the merchants of Glasgow, of the inferior quality of the pot-ash and pearl-ash supplied from the port of New-York, and having been requested by the President of the Chamber of Commerce to visit with him the stores of the inspectors of those articles in the latter city, he had collected specimens of both, of the first qualities, with a view of making some experiments.

These salts, being in their caustic state, had been placed in separate glasses, to attract water and carbonic acid from the atmosphere; and after standing ftanding feveral months, the ferruginous and earthy parts having fubfided, beautiful cryftals of the alkali were formed at the bottom of the liquor.

A folution of thefe cryftals was made in water that had been boiled fome time, to extricate its air, and precipitate fome of its earth; and to this folution of pot-afh, was added a parcel of the yellowifh, earthy matter fcraped from human teeth, which had been previoufly reduced to powder in a mortar.

Inftantly on mixing them, bubbles of air were fet loofe, and thickly floated on the furface of the mixture; and, by their long continuance without burfting, feemed to indicate a fort of tenacity in the fluid, derived probably, from animal mucilage. The coarfer part of the earthy matter foon funk to the bottom, but the finer particles took a long time to fettle; yet in a few minutes, even before the liquor had become clear, a piece of clean paper, dipped in the folution, and dried before the fire, deflagrated on being burnt, and emitted numerous flafhes and fparkles, after the manner of falt-petre; while no fuch lucid or radiant appearance was evident on fetting fire to paper that had been dried, after dipping it in a folution of the alkali alone.

It is not unworthy of remark, that the fmell of the mixture was offenfive and naufeous, refembling, as much as any thing, the difagreeable odour of ditch and puddle-water. On repeating the experiment feveral times, and in the prefence of feveral perfons, the abovementioned appearances were, with trifling variations, fimilar.

The object of inquiry having been lefs to make an entire analyfis of the lapis dentalis, or "tartar of the teeth," than to afcertain whether it contained any *feptic* acid, the author was fatisfied with the perfuafion, that the queftion is determined in the affirmative, *by the union of the feptic acid of the tartar* (which feemed to confift partly of nitrat of lime, combined with animal mucus, &c.) *with the alkali of the mixture into nitre, which deflagrated on being fubjected to the operation of fire, with the paper to which it had attached itfelf.*

In order to apply this principle, it muft be underftood what the component parts of the teeth are. Scheele and Gahn feem, as long ago as 1776, to have fucceeded in obtaining phofphoric acid from the bones of animals, by employing feptous (nitrous) acid, which diffolved their lime, and conftituted with it calcareous nitre, while the phofphoric acid was fet free. More recently, Berniard (*Journal de Phyfique, Octobre*, 1781) obtained phofphoric acid not only from foffil bones, from thofe of the whale and fea-horfe, but from the tooth of the manati, and the grinder of the elephant. It has alfo been extracted from ivory.

In fhort, thofe who experimentally attended to this fubject, have agreed, that animal *bones* are chiefly compofed of phofphoric acid and calcareous earth, or are phofphats of lime, and that their *teeth* confift principally of the like materials. Septic acid, therefore, formed in the mouth, decompounds teeth upon the fame principle that, in the experiments of the Swedifh chemifts, it difforganized bone; that is, by detaching the phofphoric acid from the lime, and combining itfelf with that earthy bafis.

Hunter (*Natural Hiftory of Human Teeth*, p. 125.) has an idea that the concretions on the teeth refemble the inteftinal balls and bezoars found in the bowels of many animals—and this opinion is probably very juft; but, perhaps, lefs juft is his other idea, that thefe extraneous matters confift merely of "earth and the common fecreted mucus." (*Difeafes of the Teeth*, p. 66.) He informs us, that he has feen fuch earthy depofitions "cover not only the whole tooth, but a part of the gums: in this cafe, there is always an an accumulation of a very *putrid* matter, and frequently a considerable tenderness and ulceration of the gums, &c."

The destruction of the enamel and bony part of the tooth, the rottenness of the alveolar processes, the ulceration, absorption, and bleeding of the gums, and the fetid breath, seem to arise occasionally from the same general cause.

After investigating the order of the chemical elective attraction subsisting between phosphoric acid, one of the constituent parts of the teeth, and other bones—and between lime, the other ingredient, and the substances with which it has a disposition to combine, Dr. Mitchill enters upon an extensive inquiry into the vital economy or animated structure of these parts.—The whole is interspersed with judicious and original remarks, accompanied with a variety of appropriate quotations from the ancient classics. But as the limits of our Journal will not admit of extending this article, we propose to resume the subject in the next Number.

---

Cit. Brugnatelli remarks that, in consequence of the rejection of Goettling's theory, he is obliged to withdraw the term *foxygen*, which he had lately given to the *azote* of the French chemists; and, instead of these, he proposes the word *septon*, derived from the Greek, and signifying putrid. This denomination was originally suggested by M. Saltonstall, and expresses, in the opinion of Brugnatelli, the essential property of azote, namely that of being the principal agent in the process of putrefaction.—The nitric acid therefore will, conformably to this change, be called by the name of *oxyseptonic*; the nitrates by that of *oxyseptonates*, &c. The author farther proposes to place the nitric acid among the productions of the animal kingdom. The French editors observe, on this occasion, to the learned Professor of Pavia, that it would now be rather a difficult attempt to maintain that division of chemical bodies into classes, according to the kingdoms of Nature.—*Annales de Chimie*, No. 86.

---

The same author communicates a method of preparing a solid gold of the finest quality, which perhaps is not unworthy the attention of the chemist, though it may not lead to medical improvements.—A solution of the nitrat of tin is precipitated by liquid sulphat of potass; the precipitate is then dried, and put into a retort with the half of its weight of sulphur, and the fourth part of the muriat of ammonia: a sulphat of tin of the brightest colour is said to be formed at the bottom of the retort. *Ibid.*

---

Van Mons, in a letter to Brugnatelli, gives the following account of some of his experiments on fulminating substances: "I am assured," says he, "that the oxyds of gold, precipitated by the oxyds of other metals, do not in themselves possess the property of fulminating; whence I conclude that some of those oxyds formed by alkalies, owe that property to the azote which is fixed in them, and which forms the combustible process (*fonction*), as is the case in the aurum fulminans. The oxyd of silver fulminates likewise with much greater force than that of gold. The grey oxyd of mercury, precipitated by ammonia, detonates by compression alone. *Ibid.*

---

M. Mussin wished to discover whether a mixture, consisting of sulphur and phosphorus, which continues in a liquid state at the temperature of the atmosphere, would not coagulate, if the proportion of the sulphur were increased. This mixture, at first, did not seem to disagree; the mass resembled wax, and showed an inclination to rise to the surface of the water, when the bottle which the author held in his hand, flew into a thousand pieces. The bursting of the bottle was accompanied with a fulmination, which resembled the report of a pistol; and a sulphureous alkaline smell filled the laboratory. The contents of the bottle which were scattered on the ground, was not inflammable; but some drops of it, when put on a mirror and pressed with the finger, took fire. The author was preserved from injury only by the circumstance that the explosion happened in the direction towards the cieling; and he imagined that this accident must be attributed to the expansion of a particular gas, which he supposes to have been the phosphorated hydrogen gas combined with sulphureous gas. *Ibid.*

---

Cit. VAN MONS communicates the following observations relative to the preparation of the black oxyds of antimony and mercury (*æthiops antimonialis*) in the humid way.

Antimony, by its combination with sulphur, or crude antimony, very forcibly retains this combustible ingredient, though it be not sufficiently saturated to part with it by simple trituration with mercury. The extinction of this metal, which was supposed to take place in the sulphur, was, after an extremely tedious experiment, so far from being accomplished, that the result appeared to be a mixture consisting of the sulphur of antimony, and black oxyd of mercury united by air. QUARIN endeavoured to remedy this defective operation by adding sulphur to the matter subjected to trituration; but this improvement tended only to abridge the labour, and afforded a still more imperfect mixture of the two sulphurs of antimony and mercury.

Van Mons proposes to prepare the antimonial æthiops by precipitating the hydro-sulphureous alkali of the antimony with the nitrat of mercury. To compose this hydro-sulphur, twelve parts of the sulphur of crude antimony are boiled with six parts of caustic potass and three parts of sulphur, in a sufficient quantity of water. In these proportions of the ingredients, the sulphur which is formed does not occasion any precipitate on cooling. A portion of the liquid is filtered, and examined whether the alkali is saturated with the antimonial sulphur. This point being ascertained, the liquor should be precipitated in a very clear and gradual manner, by means of a solution of mercury in the nitric acid.

No hydrogenated sulphureous gas is disengaged during the precipitation, as the hydrogen is converted into water by the portion of oxygen which the mercury ought to discharge, in order to become a black oxyd, this being the only degree of oxydation in which it can unite with the sulphuric base to form sulphur.

The precipitate, when washed and dried, is black, lightly tinged with green. It might be admitted into pharmacy, under the denomination of *æthiops antimonial, in the humid way*. The physicians of these departments who have prescribed it, have assured the author, that its exhibition has been attended with the best effects. *Ibid.* No. 89.

---

The same author has, in a letter to KASTELEYN, on the *non-oxygenability of the sulphuric acid*, added the following facts in addition to those stated by VAUQUELIN and BOUVIER.

1. Twenty grains of the filings of zinc, dissolved in four ounces of sulphuric acid, were distilled over the oxyd of manganese, and sufficiently diluted. During the solution, hydrogenous gas was disengaged. If the acid acid had been oxygenated, it would have imparted to the zinc its oxygen, fome of which it required to diffolve it, and the water would not have been decompofed.

2. The fame experiment made with iron inftead of zinc, afforded a fimilar refult.

3. Thirty grains of zinc, diffolved in three ounces of fulphuric acid, were digefted over the oxyd of manganefe, according to the inftructions of Giobert. The folution in this inftance took place without any difengagement of hydrogen gas; but the metal difpelled the oxygen from the oxyd of the diffolved manganefe, and not from the fuppofed oxygenated acid; it being proved by the difappearance of the red rofe colour, that the fulphuric acid owed its combination with a portion of the oxyd of manganefe, to the maximum of oxydation.

4. The fame experiment made with iron, produced a fimilar effect.

5. The nitric acid boiled with the fulphuric acid, did not oxygenate the latter.

From thefe experiments Van Mons has difcovered how to prepare a liquor for artificial bleaching, which is remarkably cheap and eafily procured. Three parts of muriatic acid muft be digefted over one part of the oxyd of manganefe, in a glafs retort clofely ftopped; after a fufficient digeftion, the limpid part is poured off and precipitated with potafs or foda, in order to feparate that portion of the oxyd of manganefe which is diffolved; the lye is then filtered, and the precipitated oxyd is added to that which is not diffolved, to wafh and make them re-oxyde in the open air, by the medium of water. In this manner the fame oxyd may continually ferve for new operations, and contribute much to the cheapnefs of the procefs.

This oxygenated liquid always retains a purple colour, if it be not furcharged with alkali; and it greatly refembles faggot-lye (*leffive de javelle*). *Ibid.*

---

M. Hildebrandt has lately made fome ufeful experiments *on the folution of mercury in the nitric acid*, of which the following is an abftract.

One hundred parts of concentrated nitric acid diffolved one hundred and twenty-five parts of mercury. The fame acid, diluted with half its quantity of water, diffolved one hundred and ninety feven parts of the metal; but when diluted with equal parts of water, it did not diffolve more than one hundred and feventy, and if mixed with double its quantity of water, its folvent power was reduced to one hundred and fifty parts.

The cryftalline form of the nitrat of mercury varies according to the circumftances in which the folution is made, whether in a low or high temperature, or if the acid is in a concentrated or weak ftate.

The nitrat of mercury uniformly depofits a portion of the white oxyd of that metal by its folution in water.

The nitrous folution of mercury forms cryftals the moment it parts with its free or unfaturated acid.

The mercurial oxyds diffolve in a fmaller quantity of the nitric acid than mercury in the metallic ftate.

The mercury is precipitated from its nitrous folution by the alkalies and cauftic earths, in colours which differ according to the manner in which the folution has been made, or according to the ftate of oxydation in which it was diffolved.

Mercury and its different combinations offer a vaft field to the inquiries and reflections of the chemift. M. Hildebrant has difcovered by his experimental rimental operations on this metal at leaſt *three hundred new facts*, and yet remarks that he has ſcarcely begun its inveſtigation. *Ibid.*

---

Van Mons has accidentally diſcovered a method of producing *a real white oxyd of mercury*, by precipitating the oxygenated muriat of mercury, formed by the mixture of the red oxyd of mercury, diſſolved in the nitric acid by heat, with a ſolution of the muriat of ſoda. The precipitates which were formed by the firſt additions of alkali, diſappeared almoſt as ſoon as they were ſeparated; yet they were every time produced in great abundance. When the whole of the acid was ſaturated with ammonia, the precipitate became fixed; and its colour was of the fineſt white, and extremely light.

Neither the cauſtic, fixed, and volatile alkalies, nor lime, affected the colour of this precipitate: which only volatiliſed to a red colour, while it was reduced; it readily diſſolved in the nitric and muriatic acids, &c. In ſhort, it appeared to be a real oxyd of mercury, from the relation in which it ſtands to the alkalies and lime. *Ibid.*

---

Dr. Scherer, of Weimar, has, in a letter to Cit. Van Mons, given the following account relative to the *extraction of ſugar from the beet-root.*

The celebrated Achard, director of the Phyſical Claſs in the Royal Academy of Berlin, has at length diſcovered a ſubſtitute for ſugar in the beet-root (*beta vulgaris*, Linn. the *runkelrübe* of the Germans); a plant which has hitherto almoſt excluſively been uſed for feeding cattle. He took twenty-five roots in their rough ſtate, weighing thirty-two pounds and a half, which, after having removed the outer rind, he bruiſed very ſmall, and expreſſed their juice. The groſs part remaining after this expreſſion was again extracted by means of hot water, ſo that both liquids, when united, weighed fourteen pounds and three-quarters. The whole was then evaporated by ſlightly boiling it in a tin veſſel, till it became of the conſiſtence of honey. During this evaporation, the impurities which ſtill remained in the juice, ſpontaneouſly ſeparated, and there is no doubt that they were carried away by the coagulation of the albumen of the roots. The juice thus inſpiſſated was then evaporated to dryneſs by a much milder heat. The reſult of this proceſs was a maſs eaſily reducible to a dry powder, of a light brown colour, which ſcarcely abſorbed any moiſture from the atmoſphere, and which had a pure ſaccharine taſte; its weight amounted to three pounds and three ounces: hence thirty-two pounds and a half of the root produced that quantity of raw ſugar.

M. Achard then made an experiment by which he proved, that one pound of the root contained only twelve drachms, or at the utmoſt two ounces of mucilaginous parts; and it is to the ſmall quantity of the latter ingredient that the eaſy ſeparation of the ſaccharine matter muſt be attributed.

In order to aſcertain the quantity of *pure* ſugar contained in a given weight of inſpiſſated juice, or *raw* ſugar, a portion of the latter was ſubjected to a ſlow heat, with a ſufficient quantity of alcohol, to diſſolve the whole of the ſaccharine ſubſtance. This ſolution, when cold, was filtered and carefully evaporated: it yielded a very white and perfectly pure ſugar. From the quantity thus obtained the following calculation reſulted; namely, that by the ordinary proceſs, eight pounds of the pureſt refined ſugar were obtained from one hundred pounds of beet-root; conſequently, that a piece of ground containing 180 ſquare yards, which could produce 46,000 pounds of roots, ought to yield twenty-two quintals of raw ſugar. By computing the produce at four pounds of the root to each ſquare foot of ground, a ſingle German mile might produce 16,756 quintals and ſeventy pounds of the root, or 134,053 pounds of white refined ſugar.

On this ſubject, Cit. Van Mons has furniſhed us with the following commentary.

"The *runkelrübe* or *dickwurzel* of the Germans is not the *beta vulgaris* of Linnæus, as it would appear from the remark of my friend, but is the *beta cicla* of that author. The ſpecific character of the latter conſiſts in its being tripetalous; while the former is polypetalous. The beta vulgaris has, according to Linnæus, no variety of white roots, as that from which Achard appears to have extracted his European ſugar; but is only a red root of a greater or leſs ſize, a yellow and thick root, and a pale green and thick root. They are ſimply diſtinguiſhed by the names of red beet-root, yellow beet-root, &c. The beta cicla is a white root with red leaves, or a white root with pale green leaves. The former is the true *runkelrübe*; and the latter is that ſpecies which the Swiſs call *mangold.*

"The runkelrübe, or the variety of the beta cicla, with leaves of a red colour, or pervaded by red veins, is the ground or field beet-root, which grows abundantly, but is very ſcarce in France. The Engliſh call it *turlip*, whence no doubt the name of turnips has originated, which has been mentioned in the news-papers as a diſcovery of Achard, ſo that they have confounded it with the thick turnip, (*braſſica laponica*), and other turnips, (*braſſica rapa*). It is therefore to be hoped that the learned director of Berlin, who excluſively devotes the laſt period of his laborious life to the improvement of botany, will ſoon remove our doubts on this ſubject.

"Margraff has formerly aſſerted that a very pure ſugar may be extracted from the freſh root of the different ſpecies of the beta cicla, by the ſame proceſs which is employed in extracting ſugar from the cane. The red beet-root, when dried and treated with ſpirits of wine, yields the twenty-ſixth part of its weight of ſugar candy, while the white beet-root gives the ſixteenth. *Vide*. Lewis's *Experimental Hiſtory of the Materia Medica*; Aikin's edition, under the article of *beta vulgaris*: and likewiſe the "*Memoires de l'Academie de Berlin*; 1749."

---

As the method of preſerving pathological ſubjects, which the ſkilful Mr. John Sheldon had adopted in his anatomical cabinet, is highly curious and intereſting, we do not heſitate to extract the following article from Profeſſor Saint-Fond's *Travels into England, Scotland,* &c.

"The anatomical cabinet of Sheldon," ſays our traveller, "contains a great variety of curious preparations. I viſited it ſeveral mornings, and examined a number of valuable deſigns made by able artiſts; but nothing in this collection intereſted me ſo much as a kind of mummy, which was very remarkable in two reſpects: firſt, on account of the ſubject itſelf; ſecondly, in regard to the manner of the preparation, and the particular care with which it had been made. It occupied a diſtinguiſhed place in the chamber where this anatomiſt uſually ſlept; and he was particularly fond of this work.

"I was introduced into a very handſome bed-room; in the midſt of which, a mahogany table of an oblong form ſtood oppoſite the bed.

"The top of the table opened by a groove, and under a glaſs frame I ſaw the body of a young woman, of nineteen or twenty, entirely naked. She had fine brown hair, and lay extended as on a bed.

"The glaſs was lifted up, and Sheldon made me admire the flexibility of the arms, a kind of elaſticity in the boſom, and even in the cheeks, as well well as the perfect prefervation of the other parts of the body. Even the fkin partly retained its colour, though expofed to the air.

"The flefhy parts, however, appeared rather dry, and there was too great a rigidity of the mufcles. This gave the figure, though it ftill poffeffed the remains of beauty, a meagre and feeble air, which confiderably diminifhed the delicacy of its traits.

"Sheldon informed me, that this was partly occafioned by the long ficknefs of which the young woman died.

"He explained to me the manner in which this preparation had been made: by injecting feveral parts of the body with ftrong fpirits of wine faturated with camphire, and mixed with a fmall quantity of turpentine.

"The fkin was prepared, and as it were, tanned with finely powdered alum, rubbed on with the hand. The inteftines were taken out, and covered with a varnifh compofed of a mixture of camphire and common refin. All the internal parts of the body underwent the fame operation, and were afterwards rubbed with alum.

"Sheldon affured me, that pulverifed camphire mixed with refin, formed an excellent compofition for preferving mufcular and fibrous parts. After having placed all the vifcera, thus prepared, in the body, he injected the crural artery with a ftrong folution of camphire, in rectified fpirit.

"Wifhing afterwards to imitate the natural tint of the fkin of the face, a coloured injection was impelled through the carotides to produce that effect.

"In this ftate the body was placed in the table aforementioned; but within a double cafe of timber. The firft is made of Virginia cedar (*juniperus Virginiana*). The inner bottom was covered with calcined chalk, to the thicknefs of one inch, in order to abforb all humidity. Upon this bed the body was placed; the box or cafe was then carefully fhut up, to fecure the body from the impreffion of the external air.

"The cafe was not opened till five years after the preparation had been made. It was then obferved to be in the fame ftate of prefervation as when it was firft enclofed. No mark of decay appeared, and no infect had introduced itfelf near the body. This fhrine had been feveral times opened before I faw the mummy; and though it then ftill poffeffed elafticity in feveral parts, it is to be fuppofed that the action of the air will ultimately wither it."

---

It gives us pleafure to mention the rapid progrefs of beneficent inftitutions in America, efpecially in thofe branches of medical practice which have a more immediate tendency to alleviate human mifery, in the lower claffes of fociety.—A lying-in ward has been lately eftablifhed in the city of New-York. The cafes which occur there are numerous enough to anfwer the purpofe of public inftruction. A courfe of lectures on the *obftetric art* is delivered every feafon, by Dr. Valentine Seamen, who at the fame time explains the anatomical, phyfiological, and practical parts of midwifery as far as is neceffary to enable *females* to exercife that profeffion with fcientific judgment; as this eftablifhment is particularly and *exclufively* devoted to their education.

---

A handfome impreffion of the anatomical appearance of the human body, after removing the common integuments, has been executed by Dr. Alexander Anderson, of New-York. Part of the abdominal and thoracic vifcera are alfo reprefented. The engraving is about thirty-fix inches by eighteen, and is executed in wood, after the manner of Mr. Bewick. This promifing young artift, who has repeatedly given proofs of his his taste and skill, has sailed for Europe, to perfect himself in the art of engraving, under the best masters in London and Paris.

### *Domestic Intelligence.*

*It having been suggested to us, that a concise Account of the different Hospitals, Infirmaries, and other Medical Institutions in Great Britain, would be acceptable to many of our Readers, and also tend to diffuse the benefits of the Healing Art; in compliance with this suggestion we request our Correspondents to furnish us with such accounts of these Establishments, as may seem likely to answer useful Purposes. We submit the following outline of the Particulars: an Account of the Origin or Foundation of the Institution: a concise History of its Progress to the present Time; a Description of its present State, with respect to Direction, Medical Officers, number of Pupils, Patients, &c. annually admitted.*

Dr. Osborn and Dr. Clarke propose to begin their lectures on the principles and practice of midwifery, and the diseases of women and children, on the first Thursday in October, at half past ten o'clock, at the house of Dr. Clarke, in New Burlington-Street, near Piccadilly: these Lectures will be continued there only, and at an hour calculated for the attendance upon the different Hospitals and the other Lectures on the different departments of medicine.

Dr. Pearson will commence his winter courses of lectures on the Materia Medica, Practice of Physic, and Chemistry, in the first week of October next, at the usual hours, at his house in Leicester-square.

Dr. Bradley will recommence his course of lectures on the theory and practice of physic, at the lecture-room, No. 102, Leadenhall-street, on Monday the 7th of October, at six o'clock in the afternoon.

Dr. Robert Kinglake, of Bristol, is preparing for the press a translation of Professor Trommsdorff's useful work, entitled "*ChemischeReceptirkunst für practische Aerzte*, &c. *i. e.* the art of writing chemico-medical prescriptions, for the use of practical physicians:—a work which cannot fail to be interesting to the practitioner in this country, as it contains almost every recent improvement in pharmacy and medical chemistry. We are persuaded that the translator will not fail to avail himself of the valuable works published by Göttling, Hahneman and Buchholz, on that subject, and especially of an anonymous publication which appeared in the year 1792, at Copenhagen, under the title "*Etwas über die Londoner Apothekerbücher*; i. e. Strictures on the different London Pharmacopœias:—we have ventured to suggest these hints, as Dr. Kinglake proposes to enhance the practical value of his Translation, by the addition of occasional notes and illustrations.

Mr. James Parkinson, of Hoxton-square, has a work in the press, which he flatters himself will be acceptable to the lovers of chemistry. It consists of *Chemical Memoranda*, systematically arranged, so as to form an analysis, or rather a compendium of modern chemistry, intended for occasional reference, with a description of the external characters of stones, minerals, &c. from Kirwan; tables of affinities from Bergman, &c. forming together a pocket remembrancer for the chemist and mineralogist.

CRITICAL

